# Patterns of experience, expression, and physiology of stress relate to depressive symptoms and self-injurious thoughts and behaviors in adolescents: a person-centered approach

**DOI:** 10.1017/S0033291723002003

**Published:** 2023-12

**Authors:** Katherine A. Carosella, Andrea Wiglesworth, Jason José Bendezú, Rylee Brower, Salahudeen Mirza, Bryon A. Mueller, Kathryn R. Cullen, Bonnie Klimes-Dougan

**Affiliations:** 1 Department of Psychology, University of Minnesota Twin Cities, Minneapolis, MN, USA; 2 Department of Psychology, The Pennsylvania State University, University Park Campus, University Park, PA, USA; 3 Center for Magnetic Resonance Research, University of Minnesota, Minneapolis, MN, USA; 4 Department of Psychiatry & Behavioral Sciences, University of Minnesota, Minneapolis, MN, USA

**Keywords:** Adolescence, cortisol, depression, MRI, person-centered, self-injury, stress responsivity

## Abstract

**Background:**

Preliminary evidence shows that discordance in stress experience, expression, and physiology (EEP) in adolescents is linked to depression, suicidal ideation (SI), non-suicidal self-injury (NSSI), and brain functioning. This study employs person-centered analysis to probe the relationship between stress responses, psychopathology, and neural patterns in female adolescents who are oversampled for engagement in NSSI.

**Methods:**

Adolescent females (*N* = 109, ages 12–17) underwent a social stress test from which self-report measures of stress experience, observer ratings of stress expression, and physiological metrics of stress (via salivary cortisol) were obtained. Multi-trajectory modeling was employed to identify concordant and discordant stress EEP groups. Depressive symptoms, SI and attempt, NSSI engagement, frontal and limbic activation to emotional stimuli, and resting state fronto-limbic connectivity were examined in the EEP groups derived from the multi-trajectory models.

**Results:**

Four groups were identified, three of which demonstrated relatively concordant EEP and one which demonstrated discordant EEP (*High Experience-High Expression-Low Physiology*). Further, replicating past research, the *High Experience-High Expression-Low Physiology* discordant group exhibited higher depressive symptoms, SI, suicide attempt, and NSSI episodes (only for sensitivity analyses based on past year) relative to other EEP groups. No significant group differences in brain functioning emerged.

**Conclusion:**

Results indicate that within-person, multi-level patterns in stress responding capture risk for dysfunction including depression and self-injurious thoughts and behaviors. Further interrogating of system-level stress functioning may better inform assessment and intervention efforts.

## Introduction

Depression and self-injurious thoughts and behaviors (SITBs), such as suicidal ideation (SI), suicide attempts, and non-suicidal self-injury (NSSI), are of great concern due to related morbidity and mortality (Fox et al., 2015; Nock, Joiner, Gordon, Lloyd-Richardson, & Prinstein, 2006). Though depression and SITBs occur across the lifespan, they frequently emerge in adolescence, a time of widespread change in the brain, body, and social landscape (Baetens et al., 2014; Giedd et al., 2009). Distal and proximal biological traits have been concurrently associated with NSSI. Alteration in the HPA axis and brain structure and function in frontolimbic regions implicated in stress and pain processing, cognitive control, and social/self representation [particularly the amygdala and medial prefrontal cortex (mPFC)] have been identified as potential biomarkers for NSSI risk (Casey, Heller, Gee, & Cohen, 2019; Kaess et al., 2021). Importantly, frontolimbic connections strengthen during adolescence (Gee et al., 2013), with the mPFC increasingly providing inhibitory control of the amygdala (reviewed by Kaess et al., 2021). Though research has associated adolescent stress responses with psychopathology (Kaess et al., 2021; Liu, Cheek, & Nestor, 2016; Stikkelbroek, Bodden, Kleinjan, Reijnders, & Baar, 2016), the role of concordance between components of the stress systems remains unclear. Recent work indicates that discordance between these systems may be crucial in identifying individuals at greatest risk of experiencing depression and SITBs (Bendezú, Thai, Wiglesworth, Cullen, & Klimes-Dougan, 2022).

### Stress experience, expression, and physiology links to depression and SITBs

#### Stress experience

An individual's perception of stress provides insights to their appraisals of themselves and stressors in their surrounding environments, which may be crucial to determining clinical outcomes (Cohen, Kamarck, & Mermelstein, 1983). Indeed, perceived stress has been positively associated with depressive symptoms (Cristóbal-Narváez, Haro, & Koyanagi, 2020), SI (Ang & Huan, 2006; Cole et al., 2015; Kleiman, Turner, Chapman, & Nock, 2018), and NSSI (Kim et al., 2015; Renteria et al., 2021).

#### Stress expression

The expression of distress has been linked to both adaptive and pathological outcomes. Expression conveys one's needs and potentially facilitates the reception of social support, thus representing adaptive response to distress (Ackerman, Abe, & Izard, 1998; Calhoun et al., 2021; Gerin, Pieper, Levy, & Pickering, 1992; Kirschbaum, Klauer, Filipp, & Hellhammer, 1995). However, heightened stress expression in adolescents has been longitudinally linked to greater risk for NSSI and SI (Kilic et al., 2023). Downplaying expression may reflect emotional competence and efforts to attain social goals (Dickerson & Kemeny, 2004; Mauss & Robinson, 2009). However, the suppression of expressed stress responses could be deleterious, as it is associated with exaggerated physiological stress responses (Schwerdtfeger & Kohlmann, 2004; Schwerdtfeger & Rathner, 2016).

#### Stress physiology

When stress is encountered, multiple physiological systems are engaged, including the hypothalamic–pituitary–adrenal (HPA) axis, which elicits the production of cortisol, increasing arousal and mobilizing resources to navigate the stressor (Herman, Ostrander, Mueller, & Figueiredo, 2005). Patterns of heightened cortisol reactivity have been found in adolescents with depression (Lopez-Duran, Kovacs, & George, 2009), though those with a history of NSSI consistently demonstrate blunted cortisol reactivity (Kaess et al., 2021). Adolescents with a history of SI may have heightened cortisol reactivity (Giletta et al., 2015); however, blunted reactivity is associated with both past and future suicide attempts (Eisenlohr-Moul et al., 2018). Alterations in HPA reactivity can at times be adaptive; heightened reactivity allows for greater sensitivity to future threat, while attenuated reactivity could protect against the deleterious effects of chronically elevated cortisol (Herman et al., 2005). However, these changes may increase vulnerability to psychopathology.

### Interplay between stress response systems

While experience, expression, and physiological (EEP) responses to stressors are independently linked to depression, SI and attempt, and NSSI, emerging research indicates that the concordance between these systems may be implicated in psychopathology (Bendezú et al., 2022). It is theorized that concordance across systems indicates healthy capacity for self-regulation, while cross-system discordance indicates disruption at the system level and thus increased risk for psychopathology (Mauss & Robinson, 2009; Selye, 1936). That said, evidence also shows that HPA reactivity acts as a moderator of stress experience and depressive symptoms [e.g. stronger positive associations observed in the presence of heightened HPA activation (Owens et al., 2019)]. As such, heightened responsivity within and across systems may index heightened risk of psychopathology.

The investigation of interplay between stress response systems has primarily employed variable-centered methods, which assess for differences on individual stress metrics between groups (Aldao, 2013). Such approaches fail to capture individual-level nuance or patterns of interaction among systems, which may be critical for linking stress to outcomes of interest (e.g. Campbell & Ehlert, 2012). As a result, some recent work has attempted to examine this cross-system interplay using multi-level, person-centered frameworks (e.g. Bendezú et al., 2021; Simon, Rab, Goldstein, Magal, and Admon, 2022). Bendezú et al. (2021) utilized multi-trajectory modeling of adolescent girls’ negative affect, positive affect, and cortisol responses to the Trier Social Stress Test (TSST) and identified four profiles. *Hyporesponsive* girls, those who exhibited higher negative affect, lower positive affect, and lower cortisol production, were at increased risk for SITBs 3 to 9 months later. Notably, low cortisol on its own was not significantly associated with these risky outcomes, suggesting a need to consider multi-system patterns in context.

Recent work has employed person-centered analyses of patterns of EEP responses, permitting the identification of varying degrees of correspondence across experience, expression, and physiology systems (Bendezú et al., 2022). Bendezú et al. (2022) examined stress responses to the TSST in adolescents diagnosed with depression and healthy controls and found three relatively concordant EEP groups and one discordant group: High Experience, High Expression, and Low Physiology (*H_experi_–H_expres_–L_physio_*). Similar to the *Hyporesponsive* profile identified in (Bendezú et al., 2021), the *H_experi_–H_expres_–L_physio_* discordant group was associated with greater depressive symptoms, SI, and NSSI engagement. Bendezú et al. (2022) also found lower frontolimbic resting-state functional connectivity (RSFC) in *H_experi_–H_expres_–L_physio_* compared to the concordant groups. These results suggest that discordance in the stress response system may incur risk for depression and SITBs, and that such risk may be explained by deficits in neurologic mechanisms involved in self-regulation. Further work is needed to examine this theory.

### The current study: aims and hypotheses

The present study aimed to further characterize the relationships among EEP correspondence, psychopathology, and frontolimbic functioning in a sample of adolescents assigned female at birth with a range of NSSI behaviors. In Aim 1, we identified participant EEP groups in response to a laboratory-based social stressor. Guided by Bendezú et al. (2022), we hypothesized that groups with varying levels of concordance would emerge. In Aim 2, we examined whether the EEP groups differed in psychopathology. We hypothesized that more discordant groups would report more severe depressive symptoms, SI and attempt, and NSSI engagement relative to the concordant groups. Finally, in Aim 3, we examined whether groups differed in neurobiological signatures associated with stress processing: mPFC-amygdala RSFC and activation of amygdala and mPFC in response to emotional stimuli. We hypothesized that more discordant groups would exhibit decreased fronto-limbic RSFC and increased amygdala and decreased mPFC activation relative to the concordant groups.

## Method

### Procedure

Adolescents (12–17 years) assigned female sex at birth (postmenarcheal) were recruited through flyers posted in the community, digital marketing, and local clinics and hospitals for a longitudinal study focused on NSSI and brain development. The study was oversampled for participants who engaged in NSSI behaviors, and participants represent a range of severity in NSSI engagement. Specifically, we aimed to recruit four equally sized groups of NSSI severity: no NSSI, mild NSSI, moderate NSSI, and severe NSSI (methods for assigning these groups are described in the online Supplement). Participants were excluded at intake for current substance use disorder, lifetime bipolar disorder or psychotic disorder, intellectual or developmental disability, major medical illness that would impact brain structure or function, or contraindications to undergoing magnetic resonance imaging. The present study focused on the first assessment wave, which included three visits consisting of (1) consent, clinical assessment, and self-report measures, (2) modified TSST, and (3) neuroimaging. This study was approved by the Institutional Review Board at University of Minnesota. See Başgöze and colleagues (2021a) for additional details.

#### Trier social stress test- modified (TSST)

EEP responses were captured in the context of a modified TSST, during which participants were asked to give an introduction speech and perform verbal arithmetic calculations while being observed by two examiners trained to remain neutral and to avoid giving reassurance or feedback (based on a validated measure of the procedure used by (Yim, Granger, & Quas, 2010; Yim, Quas, Rush, Granger, & Skoluda, 2015); adapted from the classic TSST (Kirschbaum, Pirke, & Hellhammer, 1993). This procedure has been repeatedly used to reliably elicit stress responses in female youth (e.g. Klimes-Dougan et al., 2019). Participants were scheduled to complete the TSST between 1 and 4 pm and were instructed to not eat, drink, smoke, or brush their teeth within the hour prior to providing saliva samples.

#### Experience of stress

To rate the *experience* of stress, participants were asked ‘How stressful was…’ the (1) preparation, (2) speech task, (3) math task, and (4) post-test period (e.g. ‘now’). Responses were provided on a scale from 1 (least stressful) to 5 (most stressful). This rating scale or similar rating scales have been previously employed in other studies (e.g. Gunnar et al., 2021; Klimes-Dougan et al., 2019).

#### Expression of stress

Evaluators went through a training process where they rated videos of TSST administrations to ensure reliability in ratings of expressions of stress before engaging in the ratings for this study. Evaluators were provided with a list of behavioral expressions indicative of stress, which included fidgeting, blushing, freezing, and verbal references to stress, to scaffold their responses. This rating system has been previously employed in other studies (Klimes-Dougan et al., 2019). Two examiners independently rated observed *expressions* of stress during (1) the speech and (2) arithmetic portions of the TSST from 1 (not stressed at all) to 6 (discontinued the procedure because they were showing signs of distress), and their scores were averaged together to create separate composite mean expressed stress scores for the speech and math tasks. Similar to past studies, intraclass correlations indicated that examiner ratings demonstrated moderate agreement (*ICC_speech_* = 0.701, *p* < 0.001; *ICC_math_* = 0.651, *p* < 0.001).^†^ ^1^

#### Physiological response to stress

Physiological response was captured via five salivary cortisol samples collected during the procedure: CORT1 pre-task, CORT2 immediately after the speech and math section (+15 min), CORT3 at +30 min, CORT4 at+45 min, and CORT5 at +60 min. Passive drool techniques were used and samples were stored in a −25 °C freezer until they were shipped to Universität Trier in Trier, Germany for analysis. Assay methods were consistent with Dressendörfer, Kirschbaum, Rohde, Stahl, and Strasburger (1992). Cortisol values that were >3 SDs from the grand mean in either direction (CORT1 (*n* = 3), CORT2 (*n* = 2), CORT3 (*n* = 3), CORT5 (*n* = 2)) were winsorized prior to analyses.

### Clinical measures

#### Depressive symptoms

The Beck Depression Inventory, second edition (BDI; Beck, Steer, & Brown, 1996) was used to measure current depressive symptoms. Participants rated the frequency at which they experienced 18 depressive symptoms in the past two weeks. Sum scores were computed; higher scores indicating greater depressive symptoms (skewness = 0.7, kurtosis = 2.5). When the BDI was included as a covariate in follow up analyses of our suicide ideation, suicide attempt, and NSSI models, 17-item sum scores (excluding one item that assesses suicide ideation) were used.

#### Suicidal ideation (SI)

The Beck Scale for Suicidal Ideation (BSSI; Beck, Kovacs, & Weissman, 1979) measured current suicidal and self-injurious thoughts, desires, and behavior patterns. Responses to the first five items, which were asked of all participants and only measure SI, were summed, with higher scores indicating greater SI (skewness = 1.7, kurtosis = 2.9).

#### Nonsuicidal self-injury (NSSI) and suicide attempt (SA)

The Self-Injurious Thoughts and Behaviors Interview (SITBI; Nock, Holmberg, Photos, and Michel, 2007) is a clinician-administered measure of lifetime thoughts of and engagement in SITBs. The number of suicide attempts and episodes of NSSI engagement were used for each participant. Lifetime report was used given that prior history of behavior/ideation is a significant risk factor for future engagement (Fox et al., 2015). A description of the operationalization of NSSI severity and the outcomes of these analyses can be found in the online Supplement. Additionally, sensitivity analysis for past year NSSI engagement was also conducted.

### Neuroimaging

Brain scans were conducted using a Siemens 3 Tesla Prisma scanner and a 32-channel receive-only head coil, using the Human Connectome Project (Glasser et al., 2016) multiband sequences. Structural scans were acquired using T1-weighted, multiecho Magnetization-Prepared RApid Gradient-Echo sequence and a T2-weighted Sampling Perfection with Application optimized Contrasts using different flip angle Evolution sequence. A series of functional MRI (fMRI) scans were obtained consisting of whole brain T2*-weighted functional volumes with 2 mm isotropic voxel resolution using the HCP multiband echo planar imaging sequence. Participants first underwent a resting-state scan during which they were instructed to stay awake, focus their eyes on a fixation cross, and ‘not think about anything in particular.’ Participants also completed an emotion-face matching task in which they match either emotionally expressive faces (characterized by fear or anger) or neutral shapes (Hariri, Tessitore, Mattay, Fera, & Weinberger, 2002). The emotion-face matching task was conducted via E-prime software (Schneider, Eschman, & Zuccolotto, 2012), and stimuli were projected onto a screen inside the bore of the scanner. The online Supplement contains greater information about neuroimaging data processing.

#### Resting state analyses

The CIFTI-space gray-ordinate-wise time series were used to create an average time series for each of the Glasser and Harvard-Oxford parcellations. Then, the average time series from bilateral amygdala and mPFC were extracted, cross-correlated, and Fisher's z-transformed to yield z-scores representing the RSFC between amygdala and mPFC for right and left hemispheres. The mPFC (parcellations 10v, 10r, p32, s32, 9 m, p32pr, a32pr, d32, 8BM) regions were identified using the Glasser multi-modal parcellation guide (Glasser et al., 2016). Values were averaged across hemispheres to obtain a bilateral value.

#### Task fMRI analyses

FSL FMRI Expert Analysis Tool (Woolrich, Ripley, Brady, & Smith, 2001) was used to conduct a regression analysis measuring neural activation at each gray ordinate of the brain during the emotion matching task. We included two explanatory variables (emotion face and shape conditions) of the Hariri task (Hariri et al., 2002). The produced contrast of emotion > shape z-score statistical maps in matrix form were projected back to CIFTI space for further analyses. Bilateral average z-scores for gray ordinates within amygdala and mPFC for the emotion > shape contrast were extracted for further analyses.

### Analytic approach

#### Aim 1

Following Bendezú et al. (2022), multi-trajectory modeling (Nagin, Jones, Passos, & Tremblay, 2018) was used to group adolescents based on the extent to which they exhibited similar stress EEP trajectories in response to the TSST. The PROC TRAJ procedure SAS 9.4; (SAS Institute, 2010) was utilized with the MULTGROUPS option. A nonsignificant Little's (Little, 1988) MCAR test, Χ ^2^ (123) = 63.36, *p* > 0.25, supported the use of Full-Information-Maximum likelihood to handle missing data. For expression, a linear polynomial function was estimated.^2^ For experience and physiology, quadratic and cubic polynomial functions were initially estimated, respectively. At each step of model specification (e.g. one-group, two-group), non-significant highest order polynomial functions for each stress index were systematically eliminated. Non-significant cubic and quadratic terms were systematically removed from trajectories at each step, while linear parameters were retained irrespective of significance (Andruff, Carraro, Thompson, Gaudreau, & Louvet, 2009; Helgeson, Snyder, & Seltman, 2004; Louvet, Gaudreau, Menaut, Genty, & Deneuve, 2009). The log Bayes factor approximation [2log_e_(B_10_)] was utilized at each step as a fit index. Given our sample size and recommendations from the PROC TRAJ procedure developers (Jones & Nagin, 2007; Nagin, 2005), we limited model specification to four groups. Following specification, we evaluated multi-trajectory modeling adequacy via average posterior probability (*AvePP_j_* > 0.70), odds of correct classification (*OCC_j_* > 5.00), and the ratio of the probability of subgroup assignment to the proportion of adolescents assigned to subgroups ([*Prob_j_*/*Prop_j_*]≈1) (Nagin et al., 2018). After adequacy evaluation, Wald tests were used to distinguish the groups, delineating how intercept and polynomial functions for each trajectory were relatively ‘higher’ or ‘lower’ (e.g. baseline, reactivity) across groups.

#### Aims 2 and 3

A series of regression analyses were employed to determine whether differences were observed between EEP groups in clinical and neurobiological measures of interest. In keeping with Bendezú et al. (2022), we compared each group to the *H_experi_–H_expres_–L_physio_* group in all analyses. Linear regressions were run for linear dependent variables (e.g. depressive symptoms, SI, RSFC, regional activation) and negative binomial regressions were run for count dependent variables (e.g. NSSI, SA). EEP group membership, which was dummy coded when entered into the models (with *H_experi_–H_expres_–L_physio_* left out so that it would be the default reference group), was used as the primary predictor. Age, minority race or ethnicity (as opposed to white),^3^ family income (<$ 60 000 *v.* ⩾$ 60 000), and HPA medication status (Granger, Hibel, Fortunato, & Kapelewski, 2009) were included as covariates in all analyses. Follow up analyses were conducted for SI, SA, and NSSI controlling for depressive symptoms. We checked assumptions of normality for linear outcomes prior to analysis.

## Results

Our final sample included 109 adolescent participants with complete EEP data (see Table 1 for descriptives of the study sample; sample sizes for each analysis are reported in the corresponding results table).

**Table 1.** Participant demographic and clinical descriptives disaggregated by profiles

	Total sample (*N =* 109)	*L_experi_–L_expres_–L_physio_* (*n =* 39)	*H_experi_–H_expres_–H_physio_* (*n =* 19)	*H_experi_–L_expres_–M_physio_* (*n =* 25)	*H_experi_–H_expres_–L_physio_* (*n =* 26)
Age, *M (*s.d.*)*	14.9 (1.2)	14.8 (1.3)	14.9 (1.1)	15.2 (1.1)	14.9 (1.2)
Race (non-Hispanic/ Latinx), *N* (%)					
American Indian	1 (0.9)	1 (2.6)	0 (0.0)	0 (0.0)	0 (0.0)
Asian	3 (2.8)	2 (5.1)	1 (5.3)	0 (0.0)	0 (0.0)
White	81 (74.3)	29 (74.4)	12 (63.2)	18 (72.0)	22 (84.6)
Asian/White	2 (1.8)	0 (0.0)	0 (0.0)	1 (4.0)	1 (3.8)
American Indian/White	1 (0.9)	0 (0.0)	0 (0.0)	0 (0.0)	1 (3.8)
Black/White	5 (4.6)	1 (2.6)	1 (5.3)	3 (12.0)	0 (0.0)
Native Hawaiian or Pacific Islander/White	1 (0.9)	0 (0.0)	1 (5.3)	0 (0.0)	0 (0.0)
Other (not specified)/White	1 (0.9)	1 (2.6)	0 (0.0)	0 (0.0)	0 (0.0)
Multiracial (not specified)	2 (1.8)	0 (0.0)	2 (10.5)	0 (0.0)	0 (0.0)
Race (Hispanic/Latinx), *N* (%)					
White	8 (7.3)	3 (7.7)	2 (10.5)	2 (8.0)	1 (3.8)
Black/White	1 (0.9)	0 (0.0)	0 (0.0)	0 (0.0)	1 (3.8)
American Indian/Black/White	1 (0.9)	1 (2.6)	0 (0.0)	0 (0.0)	0 (0.0)
Other (not specified)	2 (1.8)	1 (2.6)	0 (0.0)	1 (4.0)	0 (0.0)
Gross income per year, *N* (%)					
≤$24 999	8 (7.3)	3 (7.7)	2 (10.5)	2 (8.0)	1 (3.8)
$25 000–$39 999	10 (9.2)	2 (5.1)	2 (10.5)	3 (12.0)	3 (11.5)
$40 000-–$59 999	10 (9.2)	5 (12.8)	1 (5.3)	2 (8.0)	2 (7.7)
$60 000–$89 999	15 (13.8)	4 (10.3)	4 (21.1)	3 (12.0)	4 (15.4)
$90 000–$179 999	46 (42.2)	17 (43.6)	9 (47.4)	8 (32.0)	12 (46.2)
≥$180 000	20 (18.3)	8 (20.5)	1 (5.3)	7 (28.0)	4 (15.4)
Clinical characteristics					
HPA medication, *N* (%)	45 (41.3)	16 (41.0)	9 (47.4)	5 (20.0)	14 (53.8)
BDI, *M (*s.d.*)*	16.00 (14.21)	11.95 (12.24)	18.53 (15.67)	12.26 (11.94)	24.20 (14.89)
BSSI, *M (*s.d.*)*	1.42 (2.16)	1.05 (1.68)	1.79 (2.18)	0.75 (1.36)	2.36 (3.00)
NSSI history, *N* (%)	73 (67.0)	24 (61.5)	12 (63.2)	16 (64.0)	21 (80.8)
NSSI incidents, *M* (s.d.)	37.06 (79.99)	36.23 (92.20)	45.71 (102.93)	31.32 (66.12)	38.15 (56.06)
SA history, *N* (%)	34 (31.2)	8 (20.5)	5 (26.3)	6 (24.0)	15 (57.7)
SA incidents, *M* (s.d.)	1.19 (2.84)	0.92 (3.30)	0.67 (1.33)	0.44 (0.96)	2.69 (3.55)

*Note: L_experi_–L_expres_–L_physio_*, Low experience, low expression, low physiology profile group; *H_experi_–H_expres_–H_physio_*, High experience, high expression, high physiology profile group; *H_experi_–L_expres_–M_physio_*, High experience, low expression, moderate physiology profile group; *H_experi_–H_expres_–L_physio_*, High experience, High expression, low physiology profile group; BDI, Beck Depression Inventory; BSSI, Beck Scale for Suicide Ideation; NSSI, non-suicidal self-injury; SA, suicide attempt; NSSI History, SA History, The number and percentage of individuals within each group that report any history of NSSI or suicide attempt; NSSI Incidents, SA Incidents, The average and standard deviation of the lifetime number of engagements in NSSI or SA reported by each group.

### Aim 1: stress experience–expression–physiology groups

**Figure 1.**
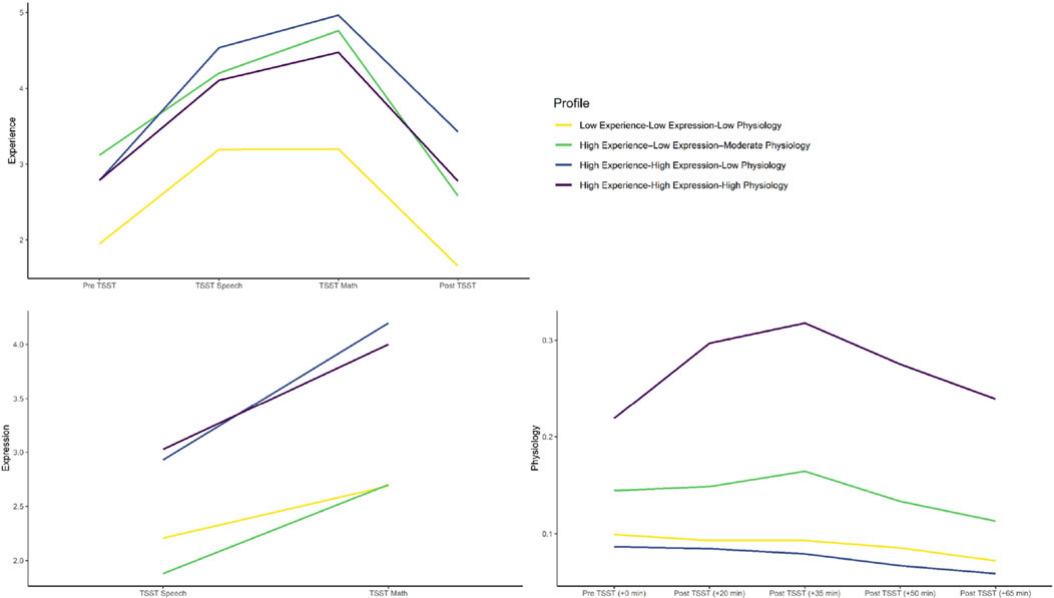
Experience, expression, and physiological trajectories of subgroups. *Note:* Physiology response was indexed by salivary cortisol (μg/dL). TSST, Trier Social Stress Test.

Multi-trajectory modeling parameter estimates, adequacy indices, and trajectory distinction analysis results are shown in Table 2. Model specification revealed four groups (Fig. 1): two- to one-group comparison [2log_e_(B_10_) = 263.66], three- to two-group comparison [2log_e_(B_10_) = 56.88], four- to three-group comparison [2log_e_(B_10_) = 10.46]. As per Nagin (2005), the final model fit the data well. With multi-trajectory modeling, subgroup trajectories may differ in intercept but not response patterning (or vice versa). In these cases, we used that significantly differing aspect to help inform our characterization of the subgroups.

**Table 2.** Parameter estimates (standard errors) and model adequacy indices for subgroups

	Stress experience	Stress expression	Salivary cortisol	*AvePP_j_*	*OCC_j_*	*Prob_j_*	*Prop_j_*	*Ratio*
Low experience - Low expression - Low physiology (*n* = 41)				0.926	37.379	0.359	0.362	0.992
Intercept	1.935* (0.158)^A^	2.238* (0.132)^A^	0.101* (0.008)^A^					
Linear	0.403* (0.049)	0.090* (0.037) ^a^	−0.001^†^ (0.001)^a^					
Quadratic	−0.028* (0.003)^a^							
High experience – Low expression – Moderate physiology (*n*=25)				0.851	17.263	0.219	0.221	0.991
Intercept	3.006* (0.228)^B^	1.908* (0.187)^A^	0.142* (0.014)^B^					
Linear	0.462* (0.064)	0.156* (0.049)^a^	0.001* (0.001)					
Quadratic	−0.032* (0.004)^a^		−0.001^†^ (0.001)^b^					
High experience - High expression - Low physiology (*n*=28)				0.926	37.724	0.252	0.248	1.016
Intercept	2.754* (0.197)^B^	2.897* (0.169)^B^	0.090* (0.009)^B^					
Linear	0.535* (0.057)	0.248* (0.047) ^b^	−0.001^†^ (0.001) ^a^					
Quadratic	−0.032* (0.003)^a^							
High experience - High expression - High physiology (*n* = 19)				0.996	828.898	0.169	0.168	1.006
Intercept	2.721* (0.212)^B^	3.018* (0.187)^B^	0.223* (0.014)^A^					
Linear	0.461* (0.067)	0.195* (0.052)^b^	0.005* (0.001)					
Quadratic	−0.030* (0.004)^a^		−0.001* (0.001)^c^					

*Note: AvePP_j_*, Average posterior probability; *OCC_j_*, Odds of correct classification; *Prob_j_*, Probability of group assignment; *Prop_j_*, Proportion of children assigned to each group; *Ratio*, Ratio of *Prob_j_* to *Prop_j_*; Upper-case and lower-case superscripts denote significant differences in intercept and polynomial estimates, respectively, within the same Trier Social Stress Test response index.

^†^*p* < 0.10 *p < 0.05.

Three subgroups emerged whose stress response groups were generally consistent with those identified in Bendezú et al. (2022): *Low Experience–Low Expression–Low Physiology* (*L_experi_–L_expres_–L_physio_*, *n* = 41), *High Experience–High Expression–Low Physiology* (*H_experi_–H_expres_–L_physio_*; *n* = 28), *High Experience–High Expression–High Physiology* (*H_experi_–H_expres_–H_physio_*, *n* = 19). A fourth subgroup emerged that was unique to our study sample; *High Experience–Low Expression–Moderate Physiology* (*H_experi_–L_expres_–M_physio_*, *n* = 25). There were no significant differences between these four groups in age, proportion of white *v.* Minority race/ethnicity youth, or gross family income (all *p*'s > 0.05).

### Aim 2: clinical correlates of stress EEP groups

As shown in Table 3, compared to the *L_experi_–L_expres_–L_physio_* and the *H_experi_–L_expres_–M_physio_* subgroups, the *H_experi_–H_expres_–L_physio_* group exhibited higher depressive symptoms and SI severity. Compared to the *H_experi_–H_expres_–H_physio_* and the *H_experi_–L_expres_–M_physio_* subgroups, those in the *H_experi_–H_expres_–L_physio_* group had higher odds of reporting greater lifetime SAs (Table 3). While there were no group differences in the episode number (severity) or in dichotomous indexes of lifetime NSSI engagement, there were some results consistent with predictions when restricting the NSSI engagement to past year. That is, those in the *H_experi_–H_expres_–L_physio_* group demonstrated significantly higher odds of greater NSSI engagements compared to those in the *H_experi_–L_expres_–M_physio_* group (Table S1).

**Table 3.** Stress Experience–Expression–Physiology profiles and clinical outcomes

Predictors	Clinical outcome							
	BDI		BSSI		Suicide attempt		NSSI	
	*n* *=* 103		*n* *=* 106		*n* *=* 109		*n* *=* 109	
	*b* (*b* se)	*B*	*b* (*b* se)	*B*	*OR*	95% CI	*OR*	95% CI
*L_experi_–L_expres_–L_physio_*^a^	**−12**.722 (3.334)*	−0.438	−**1**.243 (0.531)*^b^	−0.278	0.348	0.35–1.11	0.892	0.32–2.34
*H_experi_–H_expres_–H_physio_*^a^	−5.498 (4.017)	−0.148	−0.681 (0.632)	−0.122	0.200*	0.05–0.85	1.022	0.30–3.80
*H_experi_–L_expres_–M_physio_*^a^	−12.154 (3.871)*	−0.359	−1.444 (0.610)*^b^	−0.282	0.182*^b^	0.04–0.72	0.764	0.25–2.32
Age	2.833 (1.08)*	0.240	0.077 (0.174)	0.043	0.841^b^	0.54–1.28	2.324***	1.60–3.34
Minority race or ethnicity	3.847 (3.160)	−0.117	0.436 (0.500)	0.089	1.462	0.44–5.16	0.796	0.33–2.10
Gross family income	−4.618 (3.14)	−0.142	−0.491 (0.502)	−0.100	0.99	0.27–3.32	1.794	0.70–4.16
HPA acting medication	3.464 (2.695)	−0.120	0.897 (0.427)*^b^	0.206	1.46	0.52–4.16	1.217	0.54–2.88

*Note: b (se)*, unstandardized beta (standard error); *B*, standardized beta; BDI, Beck Depression Inventory; BSSI, Beck Scale for Suicide Ideation; NSSI, nonsuicidal self-injury; OR, odds ratio; 95% CI, 95% confidence interval; *L_experi_–L_expres_–L_physio_*, Low experience, low expression, low physiology profile group; *H_experi_–H_expres_–H_physio_*, High experience, high expression, high physiology profile group; *H_experi_–L_expres_–M_physio_*, High experience, low expression, moderate physiology profile group; Gross Family Income, below median (<$60 000) or above median (≥$60 000 = 1) (below = 0, above = 1); Suicide Attempt and NSSI are negative binomial count regression models; ^a^Reference group for effects is High Experience-High Expression-Low Physiology; ^b^Result changes in significance when covarying for suicidal ideation; * *p* < 0.05.

Results did change in some cases in follow up analyses of SI severity, SA, and NSSI that controlled for depressive symptoms (Table S2). Specifically, depressive symptoms were the only significant predictor of SI severity when incorporated into the models. While group differences in lifetime SAs between the *H_experi_–H_expres_–H_physio_* and *H_experi_–H_expres_–L_physio_* groups were maintained, differences between the *H_experi_–L_expres_–M_physio_* and *H_experi_–H_expres_–L_physio_* were no longer significant.

### Aim 3: neural correlates of stress EEP groups

Parameter estimates for our regression models are shown in Table 4. There were no significant differences between the *H_experi_–H_expres_–L_physio_* group and the other three groups with respect to amygdala–mPFC RSFC nor amygdala or mPFC activation to the emotional face-matching task.

**Table 4.** Stress Experience–Expression–Physiology profiles and neural outcomes

	Neurobiological outcome		
	Amygdala activation	mPFC activation	RSFC amygdala - mPFC
	
	*n* *=* 92	*n* *=* 92	*n* *=* 94
	
Predictors	[b (b se), *B*]		
*L_experi_–L_expres_–L_physio_*^a^	0.160(0.398), 0.052	0.164(0.218), 0.101	0.002(0.016), 0.018
*H_experi_–H_expres_–H_physio_*^a^	0.331(0.495), 0.082	0.174(0.271), 0.082	−0.011(0.019), −0.073
*H_experi_–L_expres_–M_physio_*^a^	0.431(0.465), 0.123	−0.012(0.255), −0.007	−0.002(0.018), −0.012
Age	0.212(0.129), 0.176	−0.011(0.071), −0.018	−0.013(0.005), −0.269*
Gross family income	−0.284(0.379), −0.084	−0.338(0.207), −0.190	−0.009(0.015), −0.072
Minority race or ethnicity	0.602(0.374), 0.180	0.085(0.205), 0.048	−0.006(0.015), −0.050
HPA acting medication	0.491(0.328), 0.163	0.062(0.180), 0.039	0.003(0.013), 0.029

*Note:* b (se), unstandardized beta (standard error); *B*, standardized beta; RSFC, resting state functional connectivity; mPFC, medial prefrontal cortex; *L_experi_–L_expres_–L_physio_*, Low experience, low expression, low physiology profile group; *H_experi_–H_expres_–H_physio_*, High experience, high expression, high physiology profile group; *H_experi_–L_expres_–M_physio_*, High experience, low expression, moderate physiology profile group; Gross Family Income, below median (<$60 000) or above median (≥$60 000 = 1) (below = 0, above = 1).

^a^Reference group for effects is High experience-High expression-Low physiology; * *p* *<* 0.05.

## Discussion

The present study extends evidence that discordance within EEP responses to stress are related to psychopathology and that these associations are more nuanced than can be ascertained using variable-centered approaches. Leveraging a unique sample of female adolescents at heightened risk for SITBs, we examined multi-level data (self-reported stress and psychopathology, observer reported stress, salivary cortisol, and neuroimaging) to assess whether within-person patterns across stress response indices were related to psychopathology and neural indices of activation and self-regulation. In taking a person-centered analytical approach, we identified four EEP groups: *H_experi_–H_expres_–L_physi_*, *H_experi_–H_expres_–H_physi_*, *H_experi_–L_expres_–M_physi_*, *L_experi_–L_expres_–L_physi_*. These EEP groups differed in their reports of depression, SI, suicide attempt, and NSSI (only for episodes reported in the past year), helping to further our understanding of the relationship between EEP discordance and risk for psychopathology.

The links between person-centered groups and psychopathology are illuminating; adolescents in the *H_experi_–H_expres_–L_physio_* group reported more severe depression and SI compared to those in the *L_experi_–L_expres_–L_physio_* and *H_experi_–L_expres_–M_physio_* groups and reported more frequent engagement in suicide attempts compared to those in the *L_experi_–L_expres_–L_physio_* and *H_experi_–H_expres_–H_physio_* groups. In a sample in which about two-thirds of participants had a history of NSSI engagement, no differences between groups were found in lifetime NSSI engagements, likely in part due to the pervasive shared risk factor across groups. Nevertheless, more proximal indexes of NSSI (past year) revealed group differences. Such patterns of elevated risk among youth in the *H_experi_–H_expres_–L_physio_* group are broadly consistent with previous research, which has demonstrated that high psychological stress reactivity confers risk for SI for youth with low physiological stress responsivity (Eisenlohr-Moul et al., 2018; Owens et al., 2019). Moreover, as shown here, a person-centered approach is critically important in clarifying differences for those who exhibit low physiological reactivity. Though youth in the *L_experi_–L_expres_–L_physio_* and *H_experi_–H_expres_–L_physi_* groups exhibited similar physiological reactivity, the groups were qualitatively distinct with respect to reports of psychopathology. Thus, it is likely that research that considers only one indicator of stress response in isolation obscures system-level patterns of vulnerability. Further, this person-centered approach highlighted nuance within youth who exhibited high distress during the TSST. While youth in the *H_experi_–H_expres_–H_physio_* group did not significantly differ from those in the *H_experi_–H_expres_–L_physio_* group with respect to depressive symptoms, suicide ideation, and NSSI engagement, these concordantly high responding youth did report significantly less frequent engagement in suicide attempts than those in the discordant *H_experi_–H_expres_–L_physio_* group, potentially highlighting some protective features of this concordant response profile.

Importantly, the present study provided a partial-replication and extension of the work from Bendezú et al. (2022) in a unique sample enriched for SITBs. Despite differences in samples and outcomes, the overall replication of EEP groups and associations with risk in an independent sample that is more focused on transdiagnostic risk factors for maladaptive outcomes underscores the robustness of these patterns. Conceptual replication is critical for scientific advances (Pashler & Wagenmakers, 2012). This is particularly true when employing person-centered approaches, as this approach carries with it concerns about whether identified groups are statistical artifacts or a result of over-fitting to one study sample. Our replication helps to allay those concerns, and tentatively suggests that EEP discordance may represent a reliable source of meaningful stress response heterogeneity that can be dependably captured using multi-level data and person-centered techniques such as multi-trajectory modeling.

Our study did not produce a full replication of the results from Bendezú et al. (2022). In our study, no significant differences were found between the identified EEP groups in neurobiological function. These differences in results may reflect differences in characteristics of the study samples, of which there are multiple. First, the present transdiagnostic study oversampled for a history of NSSI while Bendezú et al. (2022) employed a case-control design based on depressive disorder diagnosis. Though these clinical constructs frequently overlap, there are significant differences in the neurobiological and psychosocial mechanisms at play (Başgöze, Wiglesworth, Carosella, Klimes-Dougan, & Cullen, 2021b). Second, the present sample was made up entirely of individuals assigned female sex at birth. Bendezú et al. (2022) sample was 66% female, with adolescents in the *M_experi_–M_expres_–M_physio_* subgroup more likely to be male than female, potentially accounting for the absence of this subgroup in the current study. Additionally, the present sample was younger (mean age of 14.6 years) than that of Bendezú et al. (2022) (mean age 16.5 years). As adolescence is a time of vast neural change, particularly in frontolimbic regions (Gee et al., 2013; Power, Fair, Schlaggar, & Petersen, 2010), our hypothesized neural associations may emerge at later stages of development.

Additionally, while not considered in past research, concordance in EEP did not appear to be related to stress responsivity of the amygdala and mPFC. These findings contrast prior work that showed that left amygdala activation is related to the experience of stress during the TSST (Başgöze et al., 2021a). While it is possible that this relationship may emerge if measurements of EEP and amygdala/mPFC activation and connectivity were collected using synchronous evaluations, these results indicate a need for more work to understand the interplay between physiological and neural systems as related to stress system functioning.

## Limitations

Our results should be considered in the context of the study limitations. First, while multi-trajectory modeling analyses have been used with smaller samples (Simon et al., 2022), a larger sample would increase statistical power of our analyses. Second, there are considerations regarding the representativeness of our study sample and the generalizability of our results. This sample was majority white and higher-than-average socioeconomic status. Thus, our study failed to capture variation which may be expected due to diversity in racial and ethnic backgrounds, such as experiences of minority stress and historical oppression (Blair & Raver, 2016; Flentje et al., 2020). Additionally, the sample consisted solely of individuals assigned female sex at birth; it is important to note that stress processing differs as a function of biological sex (Taylor et al., 2002). As the majority of the present findings replicated those from a sample of both male and female youth, the results may still be generalizable. The present sample also excluded adolescents with substance use disorder, bipolar disorder, and psychotic symptoms. These exclusion criteria were intended to decrease the variability in biological metrics due to factors unrelated to NSSI, though they may limit the generalizability of findings for all engaging in NSSI. Third, SA and NSSI history as well as experience and expression of stress during the TSST were captured using single item measures. Greater validity could be achieved through more thorough probing of different aspects of these phenomena in the form of multi-item assessments (e.g. Ammerman, Burke, Jacobucci, and McClure, 2021). Fourth, this study did not capture adequate metrics of, or analyze, menstrual phase, nicotine use, or engagement in physical activity, all of which have been shown to impact HPA activity and may contribute to error in group assignment. Finally, we are unable to ascertain whether these findings index correlates or causes of risk for pathology. Considering longitudinal patterns of clinical constructs of interest will be an important next step to delineate the developmental course of EEP groups (Bendezú et al., 2021).

## Clinical implications

Our findings suggest that examining concordance across multiple levels of stress system functioning can give insight into potentially heightened risk for depression and suicidal thoughts and behaviors, illuminating points of intervention. Based on the present results, blunted physiological reactivity in the presence of heightened experience and expression of stress may represent a risk factor for psychopathology such as depression and suicidal thoughts and behaviors. Adolescents displaying EEP discordance, especially in high-risk contexts, could be targeted with interventions supporting coping and stress appraisal, which could theoretically support multi-level integration and recalibration across systems (Calvete, Orue, Fernández-González, Chang, & Little, 2020; Kallianta et al., 2021). Thus far, the limited work that has focused on modulating the cortisol response in youth has employed a vast array of measurement and treatment modalities and produced mixed findings (Slopen, McLaughlin, & Shonkoff, 2014).

## Conclusion

The present study shows evidence that multi-level, person-centered analyses of stress response patterns offer unique insight into how interplay across elements of the stress response system within groups of individuals may confer risk for, or offer protection against, psychopathology. Importantly, results indicate that the discordant group with high expression and experienced stress and low cortisol output to stress demonstrated elevated depression, suicidal thoughts, and suicide attempts. Findings advance the EEP literature by demonstrating that unique patterns of stress responses are related to nuanced patterns of risk. Thus, interventions that attend to these heightened signs of distress may be particularly useful for identifying and supporting adolescents at risk for depression and SITBs.

## Supporting information

Carosella et al. supplementary material Carosella et al. supplementary material

## Notes

1 While this study used a subset of the values from one rater, subsequent analysis showed the EEP groups were identical when ratings from both experimenters were used.

2 Although at least three time points are needed in order to define a slope in latent growth curve modeling, multi-trajectory modeling constrains slopes to be equal for all participants in a given subgroup. As such, a remaining degree of freedom is available to specify a linear slope using only two time points.

3 As this paper focuses on the stress system, the authors thought it important to include a covariate that would proxy race-related stress in our models. While we acknowledge that it is not ideal to combine all ethnoracially minoritized youth into a single group and compare them with White youth, this approach was necessary in this study to proxy of race-related stress given our limited sample size.

## Supplementary material

The supplementary material for this article can be found at https://doi.org/10.1017/S0033291723002003.
