# Supplementary material for: Patterns of experience, expression, and physiology of stress relate to depressive symptoms and self-injurious thoughts and behaviors in adolescents: a person-centered approach
Source: Psychol Med. 2023 Aug 23;53(16):7902–12. doi: 10.1017/S0033291723002003 (PMC10755230; doi:10.1017/S0033291723002003)
Supplement: Carosella et al. supplementary material [file S0033291723002003sup001.docx]

Patterns of Experience, Expression, and Physiology of Stress Relate to Depressive Symptoms and Self-Injurious Thoughts and Behaviors in Adolescents: A Person-Centered Approach

**Supplemental Materials**

**S1. NSSI Severity Group Formation**

Four groups based on both number of episodes and on severity of NSSI were created: No NSSI, Mild NSSI (fewer than four past episodes involving significant tissue damage, or unlimited NSSI episodes with no tissue damage); Moderate NSSI (four or more past NSSI episodes, with frequency less than once per month, and with significant tissue damage); and Severe NSSI (four or more past NSSI episodes, with frequency greater than once per month, and with significant tissue damage). The No NSSI and Mild NSSI groups were combined to form low NSSI and the Moderate NSSI and Severe NSSI groups were combined to form a high NSSI group for greater statistical power.

**S2. Neuroimaging Preprocessing Steps**

HCP pipeline includes three structural and four functional pipeline components. Structural pipeline components include: *PreFreeSurfer* (undistorted structural volume spaces are produced, T1 and T2 weighted images are aligned, a bias field correction and a gradient distortion correction to correct for gradient inhomogeneities within anatomical scans are applied, and each participant’s native structural volume space is registered to MNI space), *FreeSurfer* (the volume is segmented into predefined structures, including the subcortical parcels, white and pial cortical surfaces are reconstructed, and a folding-based surface registration to surface atlas is performed), and *PostFreeSurfer* (volumetric and surface based images are produced, surface registration is applied, registered surfaces are downsampled, the final brain mask and myelin maps are created). Functional data preprocessing pipelines include: *(1) fMRIVolume.* Gradient distortions within EPI images are corrected, spatial distortions are removed, volumes are realigned to compensate for subject motion, the fMRI data is registered to the structural data, the bias field is reduced, the 4D image is normalized to a global mean, and the data is masked with the final brain mask). (2) *fMRISurface.* Timeseries are brought from the volume into CIFTI space, the data are smoothed with surface-smoothing of 2mm FWHM to regularize the mapping process). (3) *ICAFIX*. Time series are linearly detrended with a high band pass filter cutoff of 2000s and a slow roll off, time series are concatenated across scans but within run and sent through independent component analysis (ICA). The FSL tool “FIX” uses the spatial independent components to classify components as “signal” or “noise”. The resulting components are then cleaned in two separate steps. First, the components are cleaned in a “non-aggressive” manner by regressing both the good and bad component time series into the data, and then the resulting bad spatial maps are multiplied by the associated time series and subtracted from the original dataset. Second, 24 head motion confounds were calculated and used as regressors to clean up all components. Third, 24 confound time series derived from the motion estimation (the 6 rigid-body parameter time series, their backwards-looking temporal derivatives, plus all 12 resulting regressors squared) are used [(Satterthwaite et al., 2013)](https://paperpile.com/c/lGYeHg/W0mDL). The motion parameters are then regressed out of the data aggressively, as they are not expected to contain variance of interest [(Griffanti et al., 2014; Salimi-Khorshidi et al., 2014; Smith et al., 2013)](https://paperpile.com/c/lGYeHg/SrP7p+UVRAI+pnFUi). *(4)* *MSMAll*. Participant data is applied for functional alignment to a group template that is based on myelin maps, resting state fMRI network and visuotopic maps [(Robinson et al., 2014)](https://paperpile.com/c/lGYeHg/aFBzZ).

Once *ICAFIX* and *MSMAll* were complete, the concatenated time series were dissociated into their respective scans and used for subsequent analyses. Following *ICAFIX* denoising and *MSMAll* alignment, CIFTI space fMRI data (both resting-state and task) was parcellated into non-overlapping ROIs using a combination of the Glasser [(Glasser et al., 2016a)](https://paperpile.com/c/lGYeHg/FKwrT) and Harvard-Oxford (Makris et al., 2006) atlases for the cortical and subcortical ROIs, respectively. Once parcellated, the CIFTI space data was converted to matrix form.

**Table S1.** Stress Experience–Expression–Physiology Profiles and additional Nonsuicidal Self-Injury outcomes.

| **Predictors** | Nonsuicidal Self-Injury | | | | | |
| --- | --- | --- | --- | --- | --- | --- |
|  | Count Past Year | | Severity | | Lifetime Engagement (Yes/No) | |
|  | *n =* 107 | | *n =* 107  (Low=45, High=62) | | *n =* 107  (No=34, Yes=73) | |
|  | *OR* | 95% CI | *b* (*b* se) | *B* | *b* (*b* se) | *B* |
| *L_experi–_L_expres–_L_physio_*^a^ | 0.698 | 0.208-2.186 | -1.090 (0.630) | 0.336 | -0.995 (0.673) | 0.370 |
| *H_experi–_H_expres–_H_physio_*^a^ | 0.271 | 0.057-1.417 | -0.809 (0.771) | 0.445 | -0.589 (0.845) | 0.555 |
| *H_experi–_L_expres–_M_physio_*^a^ | 0.250* | 0.068-0.935 | -1.210 (0.707) | 0.298 | -0.753 (0.742) | 0.471 |
| Age | 2.126*** | 1.328-3.358 | 0.510 (0.200)* | 1.664 | 0.500 (0.211)* | 1.649 |
| Minority race or ethnicity | 1.406 | 0.501-4.471 | 0.654 (0.570) | 1.924 | 1.336 (0.687) | 3.803 |
| Gross Family Income | 1.524 | 0.465-4.333 | -0.644 (0.597) | 0.525 | -0.261 (0.668) | 0.770 |
| HPA Acting Medication | 0.822 | 0.312-2.369 | 1.497 (0.496)* | 4.470 | 1.863 (0.587)* | 6.445 |

*Note:* Count Past Year = negative binomial regression restricting counts of NSSI to the past year; Severity, Low= Those in the No and Mild severity groups; Severity, High= Those in the moderate and high severity groups; *b (se)* = unstandardized beta (standard error); *B* = standardized beta; NSSI = nonsuicidal self-injury; L_experi–_L_expres–_L_physio_=Low experience, low expression, low physiology profile group; H_experi–_H_expres–_H_physio_=High experience, high expression, high physiology profile group; H_experi–_L_expres–_M_physio_=High experience, low expression, moderate physiology profile group; Gross Family Income = below median (<$60,000) or above median (≥$60,000=1) (below=0, above=1); Suicide Attempt and NSSI are negative binomial count regression models; ^a^ Reference group for effects is High Experience-High Expression-Low Physiology; * *p* < 0.05.

**Table S2.** Stress Experience–Expression–Physiology Profiles and Clinical Outcomes Controlling for the Effect of Depressive Symptoms.

| **Predictors** | Clinical Outcomes | | | | | | | |
| --- | --- | --- | --- | --- | --- | --- | --- | --- |
|  | BSSI | | Suicide Attempt | | Nonsuicidal Self-Injury ^b^  Lifetime Past Year | | | |
|  | *b* (*b* se) | *B* | *OR* | 95% CI | *OR* | 95% CI | *OR* | 95% CI |
| *L_experi–_L_expres–_L_physio_*^a^ | 0.293 (0.424) | 0.069 | 0.530 | 0.149-1.822 | 1.097 | 0.383-3.028 | 0.711 | 0.206-2.373 |
| *H_experi–_H_expres–_H_physio_*^a^ | 0.067 (0.477) | 0.012 | **0.204*** | 0.043-0.972 | 0.514 | 0.146-1.986 | 0.272 | 0.061-1.300 |
| *H_experi–_L_expres–_M_physio_*^a^ | 0.010 (0.490) | 0.020 | 0.557 | 0.107-2.948 | 0.486 | 0.151-1.555 | **0.113**** | 0.028-0.455 |
| Age | -0.113 (0.135) | -0.065 | 0.643* | 0.385-1.036 | 2.476*** | 1.752-3.488 | 2.082*** | 1.329-3.232 |
| Minority Race or Ethnicity | -0.001 (0.376) | -0.000 | 1.119 | 0.323-4.079 | 1.547 | 0.598-4.358 | 2.960* | 0.986-9.919 |
| Gross Family Income | -0.208 (0.375) | -0.044 | 1.785 | 0.466-6.629 | 1.463 | 0.795-3.462 | 1.085 | 0.323-3.276 |
| HPA Acting Medication | 0.372 (0.322) | 0.089 | 2.504 | 0.802-8.375 | 1.570 | 0.656-3.891 | 0.610 | 0.214-1.834 |
| Depressive Symptoms | 0.109 (0.013)*** | 0.721 | 1.061** | 1.016-1.114 | 1.067*** | 1.029-1.111 | 1.057** | 1.015-1.109 |

*Note: b (se)* = unstandardized beta (standard error); *B* = standardized beta; BSSI = Beck Scale for Suicide Ideation; OR = odds ratio; 95% CI = 95% confidence interval; L_experi–_L_expres–_L_physio_=Low experience, low expression, low physiology profile group; H_experi–_H_expres–_H_physio_= High experience, high expression, high physiology profile group; H_experi–_L_expres–_M_physio_= High experience, low expression, moderate physiology profile group; Gross Family Income = below median (<$60,000) or above median (≥$60,000=1) (below=0, above=1); Suicide Attempt and Nonsuicidal Self-Injury are negative binomial count regression models; ^a^ Reference group for effects is High Experience-High Expression-Low Physiology; ^b^ Note, *R* produced warnings in calculating 95% CIs for NSSI engagement, thus parameter estimates of these results should be interpreted with caution. * *p* < 0.05.
